# Quality by Design–Based Assessment for Analytical Similarity of Adalimumab Biosimilar HLX03 to Humira®

**DOI:** 10.1208/s12248-020-00454-z

**Published:** 2020-05-08

**Authors:** Erhui Zhang, Liqi Xie, Peilan Qin, Lihong Lu, Yanpeng Xu, Wenyuan Gao, Linlin Wang, Michael Hongwei Xie, Weidong Jiang, Scott Liu

**Affiliations:** Shanghai Engineering Research Center of Anti-tumor Biologic Drugs, Shanghai Henlius Biotech Inc., Shanghai, China

**Keywords:** adalimumab biosimilar, analytical similarity assessment, CQA, QbD, ADCC

## Abstract

Quality by design (QbD) is an efficient but challenging approach for the development of biosimilar due to the complex relationship among process, quality, and efficacy. Here, the analytical similarity of adalimumab biosimilar HLX03 to Humira® was successfully established following a QbD quality study. Quality target product profile (QTPP) of HLX03 was first generated according to the public available information and initial characterization of 3 batches of Humira®. The critical quality attributes (CQAs) were then identified through risk assessment according to impact of each quality attribute on efficacy and safety. The anticipated range for each CQA was derived from similarity acceptance range and/or the corresponding regulatory guidelines. Finally, a panel of advanced and orthogonal physicochemical and functional tests and comparison of 6 batches of HLX03 and 10 batches of the reference standard demonstrated high similarity of HLX03 to Humira®, except for slightly lower percentage of high mannosylated glycans (%HM) in HLX03 which had no effect on FcγRIII binding and antibody-dependent cell-mediated cytotoxicity (ADCC) activity in human peripheral blood mononuclear cell (PBMC). All above demonstrated the feasibility and efficiency of QbD-based similarity assessment of a biosimilar monoclonal antibody (mAb).

## Introduction

Development of biosimilars will lead to more affordable biological treatments and increase patient access to otherwise expensive therapies. It is especially important for therapeutic mAbs, which represents a major cost burden for health-care providers on rapidly increasing usage (1). Because of the complexity of antibody structure as well as its sensitive nature to the manufacturing process, the development of biosimilar faces great technical challenges and risks of not being similar.

Quality by design (QbD) is a modern approach for the development of recombinant protein therapeutics that can both reduce risks to patients and streamline the developmental path based on risk analysis and sound sciences (2). The quality is predefined and built into a product with the knowledge of risk assessment and management involving in the manufacturing, storage and distribution of the product and on how to best mitigate potential risks (3,4). With the advantage of introducing more flexibility into the manufacturing process, reducing developmental cost, as well as decreasing the regulatory burden from QbD approach, marketing authorization applications integrated with QbD are encouraged by regulatory agents such as European Medicines Agency (EMA), US Food and Drug Administration (US-FDA), and China National Medical Products Administration (NMPA) (2,5).

QbD approach is especially suitable for biosimilar development. The quality attributes and criteria identified from the originator reference standard (RP) in advance can make the process target-oriented and reduce the risks of not being similar. In addition, since the product properties have already been defined by the RP, QbD process can be simplified and more feasible in determination of quality target product profile (QTPP) and critical quality attributes (CQA) (6,7). As recommended by FDA “abbreviated” development program 351 (k), the establishment of biosimilarity is critical for a biosimilar development, and to achieve analytical similarity is the key to the reduction of expenses and duration of the clinical studies (6,8,9). Similarity studies are much more convincing to agency reviewers when QbD principles are incorporated into the development (5).

**Chart 1:**
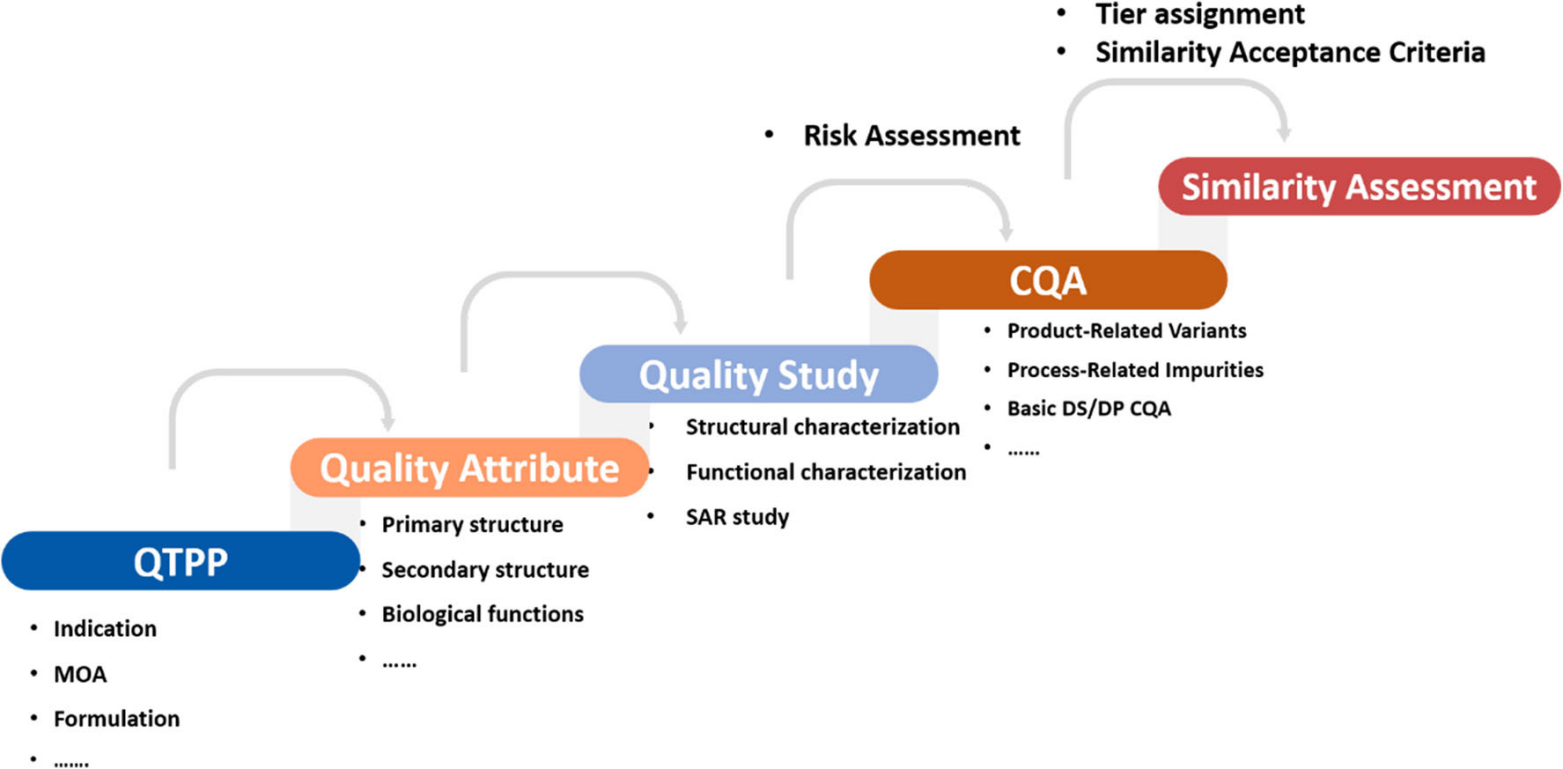
Flow chart of analytical similarity assessment process following a QbD strategy

Analytical similarity assessment is the first and most critical step for success of a biosimilar development, and its main purpose is to identify and evaluate the similarity of all quality attributes that could impact purity, potency, safety, and efficacy (10). By following QbD guidelines, a quality study platform for analytical similarity assessment as shown in Chart 1 was established in our company. The biosimilar program requires a clear definition of the QTPP in the beginning, and the physicochemical and functional properties are determined from RP characterization. Proper analytical methods that can sensitively and specifically detect and characterize the differences of product quality attributes are selected for analytical testing thereafter. Next, these quality attributes are ranked and filtered according to their risks to potentially impact biological activity, pharmacokinetics/pharmacodynamics (PK/PD), safety, efficacy, and immunogenicity to identify the CQAs. After that, each quality attribute is assigned to one of three tiers of criticality category representing high, moderate, or low risk, and corresponding statistical methods are applied among three tiers with appropriate similarity acceptance criteria (11). Once the similarity acceptance criteria were established, the similarity of each quality attribute between a biosimilar and the RP is evaluated accordingly. Any differences detected in the quality attributes will have to be appropriately justified with regard to their potential impact on safety and efficacy.

HLX03 was developed by Henlius as a proposed adalimumab biosimilar to Humira®. Adalimumab works as a steric hindrance molecule to block the association of tumor necrosis factor α (TNFα) to cell surface receptor by binding and neutralization TNFα (12). The humanized IgG1 antibody was approved by NMPA to treat patients with rheumatoid arthritis (RA), psoriatic arthritis (PA), or plaque spondylitis (Ps) (13). In this study, an example on how to implement QbD philosophy is provided for the analytical similarity assessment of HLX03 to Humira® licensed in China (CN-Humira®). Only NMPA approved indications are included in the QTPP list of HLX03. Similarity analysis data was assessed by tier-based criteria according to risk assessment results of corresponding quality attributes after a QbD quality study. Briefly, primary mechanism of action (MOA) related attributes are considered and assigned as the highest risk tier 1; while quality attributes that have little impact on clinical effect of RA, PA, and Ps, such as high mannosylated glycans (HM) and antibody-dependent cell–mediated cytotoxicity (ADCC), were categorized as low-risk tier 3 quality attributes. The other HLX03 quality attributes with potential risks in-between are assigned as tier 2 for analytical similarity assessment. The study demonstrated that HLX03 is highly similar to CN-Humira® in terms of physicochemical properties and biological activities, suggesting similar product quality, efficacy, and safety.

## Materials and Methods

### Materials

Ten batches of CN-Humira® (40 mg/0.8 mL pre-filled syringe) were purchased from AbbVie. Six lots of HLX03 (40 mg/0.8 mL/vial) were manufactured by Shanghai Henlius Biopharmaceuticals, China. Each sample was stored in multiple aliquots and at − 80°C before expiration to avoid repeated freeze/thaw cycles. Chemicals were purchased from Sigma without special mention. Data of HPLC and UPLC were acquired and processed by Empower™ 3 (Waters) software without special mention. The primary structure, higher-order structure, purity/impurities, particles, Fc receptor binding assays, and forced degradation samples are analyzed as previously described (14).

### Fc-Effector Function Bioassays

Fc-effector functions were evaluated by detecting the cytotoxicity in the presence of HLX03 or CN-Humira® *in vitro*. ADCC was measured by colorimetric luciferase release assay. Briefly, TmTNF_CHO-S cells (transmembrane TNFα expressing Target cells, Henlius, 1 × 10^4^ cells/well) and NFAT_CD16 Jurkat cells (Effector cells, Henlius, 5.0 × 10^4^ cells/well) were seeded in a 96-well plate. A 1.5-fold serially diluted mAbs at a concentration of 4000–104.049 ng/mL were added. After incubation at 37°C, 5% CO_2_ for 6 h, the cell cytotoxicity was measured by quantification of released luciferase signal intensity and reported as ADCC activity. To measure complement-dependent cytotoxicity (CDC) activity, TmTNF_CHO-S cells were incubated with 3-fold serially diluted mAbs (1000 μg/mL~50 ng/mL) in the presence of normal human serum as a complement source. After incubation for 4 h at 37 °C in 5% CO_2_, cell viability through reading the absorbance at 450 nm (OD450) with CCK8 reagent (Dojindo) was measured to obtain CDC activity results. Relative potencies of both ADCC and CDC were determined using parallel logistic assay (4-parameter fit) by comparing the EC50 of the sample to an in-house reference material.

### PBMC-Based Bioassay

The FcγRIIIa-binding affinity and ADCC activity of HLX03 and the RPs were further analyzed in human peripheral blood mononuclear cells (PBMC). PMBC cells (Allcells) were incubated with mAbs with a 2-fold serial dilution for 30 min followed by TNFα-Biotin (Sinobio) incubation. After labeled by Streptavidin-PE (eBioscience), PBMC cells were analyzed by BD Accuri C5 cytometer instrument. The results were analyzed by SoftMax software using four-parameter fitting model, and the FcγRIIIa-binding activity was evaluated by comparing the EC50 of the test sample to that of a reference. For ADCC activity assay, TmTNF_CHO-S cells and LPS-induced monocyte were incubated with mAbs at different concentration, respectively. The expression of tmTNF-α on the LPS stimulated monocytes was confirmed by flow cytometry. PBMC cells were co-cultured with mAbs-treated cells for 5 h, and then the cytotoxicity of HLX03 and the RPs were measured by CytoTox 96® Non-Radioactive Cytotoxicity Assay kit (Promega).

### Anti-apoptosis Assay

The TNFα could induce apoptosis of human myeloid leukemia cell line U-937 cells with increased activity of Caspase 3/7, which could be blocked by adalimumab. The anti-apoptosis bioassay measured the TNFα-induced activity of Caspase 3/7 on U-937 cells (National Infrastructure of Cell Line Resource, China).

### TNFα-Binding Assay

Binding affinity of HLX03 and CN-Humira® to soluble TNFα was measured by both surface plasma resonance (SPR) and a cell-based ELISA assay. In the cell-based–binding assay, the TmTNFα_CHO-S cells were incubated with mAbs with a 3-fold serial dilution at a concentration from 66,667 to 0 ng/mL for 30 min followed by FITC-labeled Goat-anti human secondary antibody (Abcam) incubation. The unbound rates were then analyzed by BD Accuri C5 cytometer instrument. The tmTNFα-binding affinity was determined by EC50 as described above.

### TNFα Neutralization Assay

Inhibitory activity of adalimumab on the cell-killing ability of soluble TNFα was measured through the TNF neutralization assay using a mouse fibroblast L-929 cell line (ATCC). Briefly, L-929 cells were treated with actinomycin D, followed by TNFα incubation with different concentrations of mAb samples. The neutralization ability of mAbs was determined using MTS assay by comparing the EC50 of the sample to a reference.

### Suppression of ICAM Expression Assay

The expression of intercellular adhesion molecule 1 (ICAM1) of human umbilical vein endothelial cell (HUVEC, Allcells) could be induced by both LTα and TNFα. The suppression ability of adalimumab on ICAM1 expression was measured by ELISA as previously described (15). The HUVEC cells were cultured with medium containing TNFα and different concentrations of adalimumabs. The HUVEC cells cultured with LTα and adalimumab were used as control.

### Forced Degradation Studies

The degradation profiles of HLX03 and CN-Humira® were determined under the stress circumstances of light (4500 ± 500 lx) exposure, strong acid (pH 4), strong alkali (pH 10), shaking at speed of 1000 rpm at 25°C, strong oxidizer (1.0% tBHP) at 4°C, and higher temperature (50°C), and the stress conditions and sampling points were similar to a previous study (14). Subsequent characterization of the forced degradation samples was conducted by using a panel of stability-indicative methods, including LC-MS, CEX, SEC, CE-SDS, and HER2-binding assay.

## Results

According to NMPA, EMA, FDA, and ICH regulatory guidelines for the development of biosimilar products (3,11,16–18), a stepwise approach incorporating QbD was used to evaluate the similarity of HLX03 to CN-Humira®. A total of 6 batches of HLX03 and 10 batches of CN-Humira® were characterized to compare their primary structure, higher-order structure, charge heterogeneity and post-translational modifications, glycosylation, molecular size heterogeneity, immunological properties, biological activity, as well as forced degradation behavior by a series of state-of-the-art and orthogonal analytical methods. The analytical similarity study was performed following a QbD approach, from establishment of QTPP, selection of analytical technologies and methods, risk assessments to determine CQAs, to the establishment of acceptance criteria for the assessment of analytical similarity.

### QTPP List Establishment

The first step in the QbD approach for biosimilar development is the establishment of the QTPP. Following the ICH guideline Q8 (R2) and an example published by Nadja Alt *et al.* (19), indication, primary MOA and relative impact factors, route of administration, dosage form, dosage strength, container closure system, and drug product quality criteria were included in the QTPP list of HLX03 (Table I). Based on the clinical, pharmacokinetic and physicochemical characteristics of Humira®, as well as publicly available information of CN-Humira®, the contents of QTPP items were determined. Only NMPA authorized indications (RA, PA, and Ps) were included in the QTPP list.

**Table I:** QTPP list of HLX03 as a Biosimilar of CN-Humira®

Attribute	Target
Indications	Rheumatoid arthritis (RA)
	Ankylosing spondylitis (AS)
	Plaque psoriasis (Ps)
Primary MOA	Neutralization of soluble TNFα
Critical features impacting MOA	TNFα binding
Dosage form	Liquid for subcutaneous injection
Dosage strength	40 mg/0.8 mL/vial
Route of administration	Intravenous administration
Product quality	Compliant to Chinese pharmacopoeia
Purity/impurities	Acceptable patient risk, compliant to Chinese pharmacopoeia
Shelf-life of drug product	24 months at 2~8°C
Inner packaging materials of drug product	Single use glass vial, rubber stopper, aluminum cover

### Collection of Quality Attributes and QbD-Based Analytical Methods Development for HLX03

Quality attributes were collected based on the structure, MOA, efficacy and safety of adalimumab, as well as the initial characterization of 3 batches of the RP at the beginning of the development. Physicochemical properties (the primary, secondary, tertiary, and quaternary structures; posttranslational modifications), purity and product variants, impurities, and functional properties (immunochemical properties and bioactivities) were included in the list of quality attributes. All possible biological functions of adalimumab, even those reported to be clinically irrelevant to the RP, such as ADCC and CDC (12), were included in the list for similarity assessment (Table II).

**Table II:** Analytical similarity comparison of selected quality attributes of HLX03 to the RP

Test classification		Quality attributes	Methods	Risk	Tier	Similarity assessment
Physicochemical properties and purity	Primary structure	Amino acid sequence	Reduced peptide mapping after being digested by trypsin and chymotrypsin, respectively (reduced LC-UV/MS/MS)	High	3*	Identical
		Molecular weight	Intact protein molecular mass analysis (LC-UV/MS)	Moderate	3*	Same mass species
			Reduced protein molecular mass analysis (LC-UV/MS)	Moderate		Same mass species
			Papain digested protein mass analysis (LC-UV/MS)	Moderate		Same mass species
		Disulfide linkage	Non-reduced peptide mapping after tryptic digestion (non-reduced LC-UV/MS/MS)	High	3*	Identical
		Free thiols	Free thiol fluorescent detection kit	High	3^#^	Similar
		Post translational modifications	Reduced peptide mapping after tryptic digestion (reduced LC-UV/MS/MS)	Moderate	3^#^	Same site, similar occupancy
		Glycosylation site	Deglycosylated and reduced peptide mapping (deglycosylated and reduced LC-UV/MS/MS)	High	3*	Identical
	Higher order structure	Secondary and tertiary structure	Differential scanning calorimetry (DSC)	Moderate	3*	Similar
			Circular dichroism (CD)	Moderate	3*	Similar
			Fluorescence spectroscopy (FLR)	Moderate	3*	Similar
	Charge variants	Acidic peaks	Cation exchange chromatography (CEX), and imaged capillary isoelectric focusing (icIEF)	Moderate	2	Similar
		Main peak		Moderate	2	Similar
		Basic peaks		Moderate	2	Similar
		Isoelectric point	Imaged capillary isoelectric focusing (icIEF)	Low	3*	Identical
	Size Variants	Low-molecular-weight fragments	CE-SDS (Reduced and Non-reduced)	High	2	Similar
		High-molecular-weight aggregates	SEC-HPLC-UV	High	2	Similar
			SEC-HPLC-MALS	High	3*	Similar
		Monomer	SEC-HPLC	Moderate	2	Similar
	Glycosylation	Site Occupancy (NGHC)	Reduced CE-SDS	High	2	Similar
		Afucosylation	HILIC UPLC-FLD	Low	3^#^	Similar
		Galactosylation		Low	3	Similar
		High mannose		Low	3	Lower in HLX03, no meaningful clinical impact
		Sialic acid	HILIC UPLC-FLD	Moderate	3^#^	Similar
			HPLC-FLD	Moderate	3^#^	Similar
Functional properties	Immunochemical properties	FcRn	SPR analysis of molecular interaction between immobilized recombinant human FcRn/FcγRs and mAbs	Moderate	2	Similar
		FcγRIa		Low	3	Similar
		FcγRIIa		Low	3	Similar
		FcγRIIb/c		Low	3	Similar
		FcγRIIIa (V)		Low	3	Lower in HLX03
		FcγRIIIa (F)		Low	3	Lower in HLX03
		FcγRIIIb		Moderate	2	Lower in HLX03
		FcγRIII	Flow cytometry analysis of FcγRIII binding to PBMC	Low	3	Similar
		C1q	ELISA	Low	3	Similar
	Bioactivity	Soluble TNFα binding	ELISA	High	1	Similar
			SPR	High	1	Similar
		Neutralization of Soluble TNFα	Cell-based assay	High	1	Similar
		Membrane type TNFα binding	Cell-based assay	Low	3	Similar
		TNFα induced inhibition of ICAM-1 expression	Cell-based assay	Low	3	Similar
		LTα induced inhibition of ICAM-1 expression	Cell-based assay	Low	3	Similar
		Anti-Apoptosis	Cell-based assay	Low	3	Similar
		ADCC	Reporter gene assay use tmTNF-α overexpressed CHO-S cell line as target cell and FcγRIIIa overexpressed Jurkat T cell line as effector cell	Low	3	Lower in HLX03
			*In vivo* simulating assay use tmTNF-α overexpressed tmTNFα_CHO-S cells as target cell and PBMC as effector cell	Low	3	Similar
			Clinically representative assay use LPS stimulated monocyte cell as target cell and PBMC as effector cell	Low	3	Similar, both negative
		CDC	Cell-based assay	Low	3	Similar
Process-related impurities		DNA	qPCR	High	3^#^	Similar
		HCP	ELISA	High	3^#^	Similar
		Protein A	ELISA	High	3^#^	Similar
Strength		Concentration	A280	High	2	Similar
Particles		Sub-micron particles	Dynamic light scattering (DLS)	Moderate	3	Similar
		Sub-visible particles	Micro-flow imaging (MFI)	Moderate	3	Similar

Tier 3* is assigned because the nature of the assay is qualitative despite of “High” or “Moderate” risk ranking;

Tier 3^#^ is assigned because the low amount of analyte despite of “High” or “Moderate” risk ranging

Sensitive and fit-in-purpose analytical methods were developed to analyze the selected quality attributes thereafter. During QbD-based analytical development, a prospective summary of the desired characteristics of analytical methods were provided. Scientifically sound, reproducible, and reliable methodologies that can maximize the potential for detecting differences between HLX03 and the RP were preferred. Critical method parameters, which had a high probability of impacting the ability to meet system suitability criteria and/or the reported values, were carefully qualified or verified for the intended use. As show in Table II, a series of high-sensitivity and orthogonal test methods were designed and developed accordingly. The specific properties of analytical methods (specificity, sensitivity, precision and potential limitations, etc.) were justified by method qualification or verification.

### Establishment of CQA List to Ensure the QTPP and Product Quality

The QTPP and additional sources of product quality attributes are the basis for assembling a list of potential CQAs. Criticality of quality attributes was assessed using a risk ranking and filtering (RRF) approach developed by Roche/Genentech (19) to evaluate relevance of each attribute to the clinical outcomes, following risk assessment principles set forth in the ICH Quality Guidelines Q8 and Q9. The RRF approach incorporates two factors: impact and the uncertainty of the impact. The impact and uncertainty scores were determined based on thorough scientific literature investigation and in-house experiments if possible. The two scores are then multiplied to generate a risk score, which is filtered and evaluated to identify final CQA.

The amino acid sequence, disulfide linkages, size variants, acidic charge variants, glycosylation, and process-related impurities were identified as CQAs for HLX03. Amino acid sequence variants and mismatching disulfide linkages might lead to conformational and functional change of adalimumab. High molecular weight species (aggregates) in general have higher immunogenic potential compared with the monomer, while the low molecular weight species (fragments) may cause loss of efficacy (20). Acidic charge variants containing sialic acid may weaken the ADCC activity and potentially induce the anti-drug antibodies (21). Adalimumab is N-glycosylated at Asn 301 in the constant region of each heavy chain (HC). The glycan heterogeneity is related to Fc effector functions, stability, pharmacokinetics, and antigenicity of mAbs (22). Since Fc-related bioactivity has little contribution to the observed clinical effect on RA, PA and Ps (12), afucosylated, high mannosylated, and galactosylated types of glycans were identified as none-CQA for HLX03. In the meantime, glycan species containing N-glycolylneuraminic acid (NGNA) that can cause potential immunogenicity was treated as a CQA. Because the deglycosylated IgGs exhibited higher aggregation rates, non-glycosylated heavy chain (NGHC) was considered as a CQA. Although low uncertainty scores were assigned, the process-related residuals listed in Table II were all identified as CQAs due to the increased toxicity and/or potential immunogenicity.

### Tiering of the Quality Attributes for Analytical Similarity Assessment

Attributes/assays were assigned to 1 of the 3 tiers based on risk assessment as shown in Table II. Following FDA regulatory guideline (11), each tier was associated with a specific methodology of statistical analysis plan for similarity assessment. Tier 1 is reserved for the most critical attributes/assays with direct impact on the primary MOA. Equivalent test is applied for tier 1 attributes. An interval (− 1.5SD, 1.5SD) that can support 90% confidence interval is recommended in two one-sided tests to determine the similarity of HLX03 to the RP. The attributes/assays with moderate to high criticality were categorized as tier 2. Tier 2 attributes/assays were considered to be similar when 90% of the HLX03 lots fell within a pre-defined quality range of mean ± 3 standard deviations of the RPs. Tier 3 attributes/assays included those with the lowest risk to clinical outcomes. In particular, tier 3 was also assigned to the qualitative or trace amount attributes/assays, despite of “High” or “Moderate” risk ranking. Similarity of tier 3 attributes was determined through visual comparison.

### Analytical Similarity Comparison of Physicochemical Properties, Purity, and Impurity Profiles of HLX03 to the RP

**Fig. 1:**
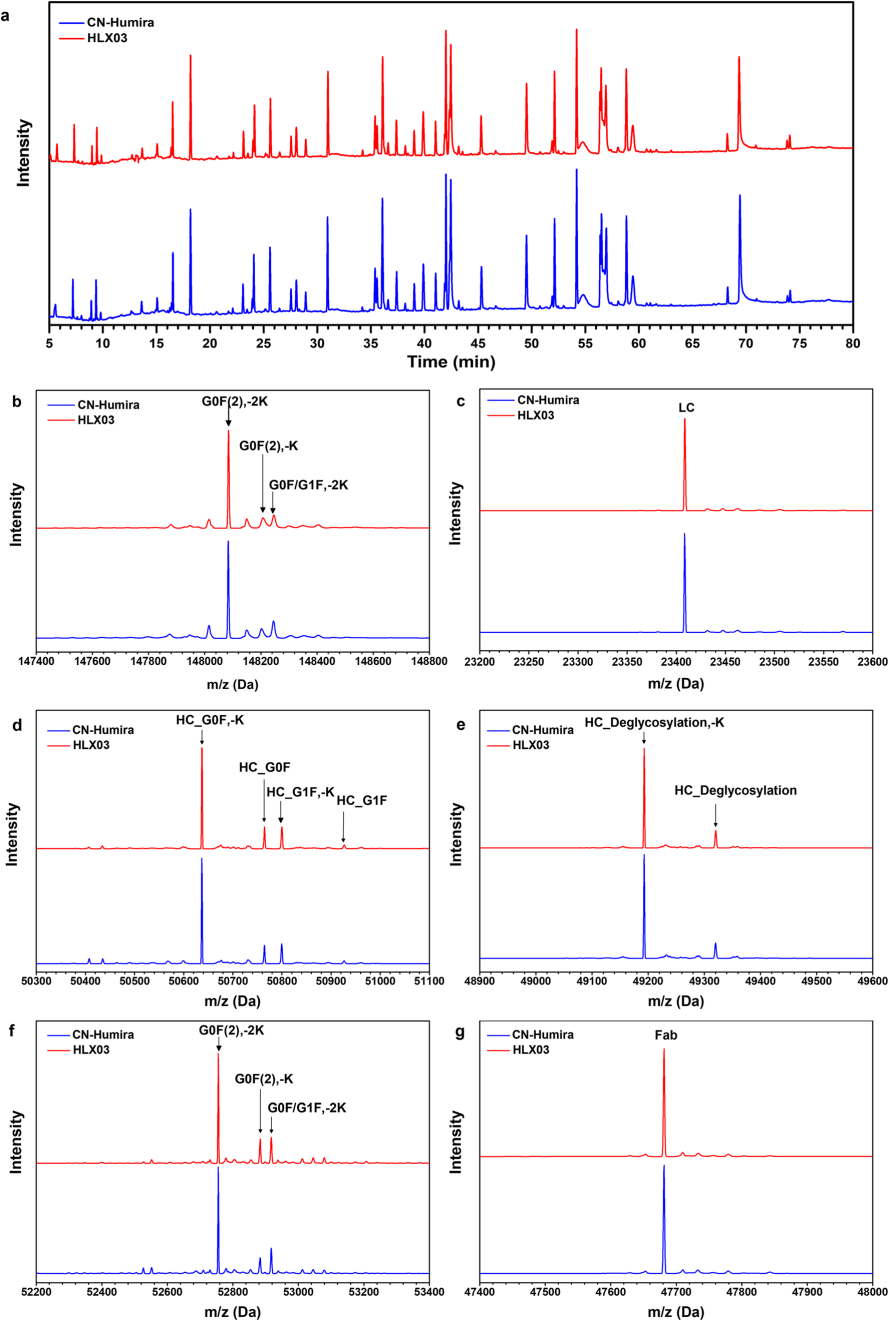
Primary structure of HLX03 and CN-Humira®. **a** Tryptic peptide maps; MS spectra of **b** Intact mAb, **c** reduced LC, **d** reduced HC, **e** reduced and deglycosylated HC, **f** Fc fragment, and **g** Fab fragment

**Fig. 2:**
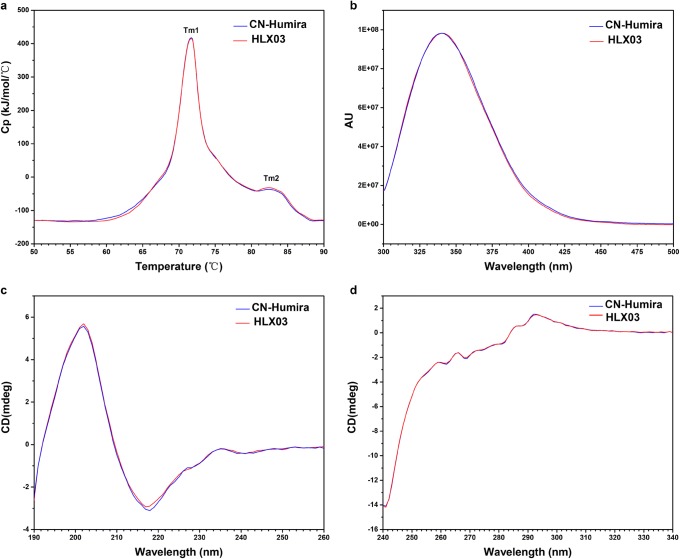
Higher-order structures of HLX03 and the RP. **a** DSC, **b** FLR, **c** far‐UV circular dichroism (CD) spectra, and **d** near‐UV CD spectra

HLX03 has the same primary structure and highly similar higher order structures to the RP. The primary structure of HLX03 and CN-Humira® was compared and determined by LC-MS analyses of protein masses, reduced peptide mapping, disulfide linkage analysis, and free thiol measurement. Both HLX03 and the RP are consistent with 100% coverage of theoretical protein sequence of adalimumab, demonstrated by peptide maps of alternative tryptic and chymotryptic digests (Fig. 1a). Same posttranslational modification types and sites, as well as highly similar site occupancies, were determined among all the products by LC-MS/MS tryptic peptide mapping. Same mass species were observed from LC-MS analyses of intact, reduced, deglycosylated and reduced, and papain-digested fragments of HLX03 and the RP, and closely matched the corresponding theoretical molecular weights of adalimumab (Fig. 1b–g). Non-reducing peptide maps and the total number of free thiols in all the mAb products demonstrated IgG1 subtype of disulfide pattern and no scrambling of disulfide linkages in both HLX03 and the RP. The higher-order structures, which are vital for bioactivity, safety and stability, are highly similar between HLX03 and the RP as demonstrated by differential scanning calorimetry (DSC), circular dichroism (CD), and fluorescence detection (FLR) (Fig. 2).

**Fig. 3:**
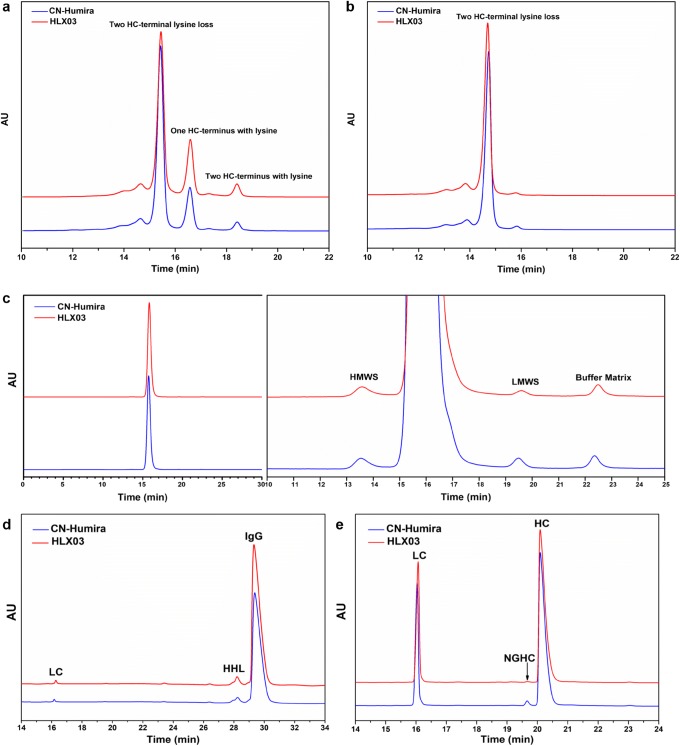
Comparison of charge and size variant profiles of HLX03 and CN-Humira®. **a** CEX chromatograms before CpB digestion, **b** CEX chromatograms after CpB digestion, **c** SEC chromatograms with enlarged version in the right panel, **d** non-reduced CE-SDS chromatograms, and **e** reduced CE-SDS chromatograms

HLX03 showed similar high level of purity as well as comparable charge and size variant profiles to the RP, except for slightly higher level of basic variants due to unprocessed heavy chain (HC) C-terminal lysine and slightly lower level of non-glycosylated HC (NGHC) size variant. The charge variants were compared by both imaged capillary isoelectric focusing (icIEF) and cation exchange chromatography (CEX). LC-MS characterization of the CEX fractions revealed that the basic peak BP1 was resulted from unprocessed C-terminal lysine on one of the HCs, and BP2 was resulted from unprocessed C-terminal lysine on both HCs. The C-terminal lysine residues can be rapidly cleaved by endogenous carboxypeptidase B (CpB) *in vivo* (23) and considered as a non-CQA for HLX03. After the removal of C-terminal lysine by CpB, HLX03 exhibited similar levels of basic and acidic variants to the RP (Fig. 3a, b). The size exclusion chromatography (SEC) and capillary electrophoresis-sodium dodecyl sulfate (CE-SDS) profiles were highly similar between HLX03 and the RP, and no unique species were detected in any of the products (Fig. 3c–e). Reduced CE-SDS profiles indicated relatively lower NGHC in HLX03 batches (Fig. 3e). The particle sizes of active pharmaceutical ingredient and sub-visible particles were highly similar too between HLX03 and the RP, compared by dynamic light scattering (DLS) and micro-flow imaging (MFI). The process-related impurities (residual DNA, protein A, and HCP) were all at similarly low levels in the products. Highly similar forced degradation behavior and trends were observed for HLX03 and the RP under higher temperature, illumination, acidity, alkalinity, oxidation, and shaking stress conditions.

Same glycosylation site and glycan types were determined between HLX03 and the RP by reversed-phase and hydrophilic interaction (HILIC) LC-MS/MS analyses, and comparable glycan profiles were observed by HILIC UPLC-FLD. The levels of G0F, galactosylated, afucosylated, and sialylated glycans were all similar in the tested products, except that HLX03 has lower proportion of high mannosylated glycans (%HM) than the RP (2.5% *vs* 8.0%) (Table III). The impact of the difference was further evaluated in the following biological activity comparison.

**Table III:** Comparison of glycans between HLX03 and CN-Humira®

Sample	G0F%^*^ mean (range)	HM%^§^ mean (range)	Sialylation%^†^ mean (range)	Gal%^‡^ mean (range)	Afuc%^**^ mean (range)	NANA mol/mol mean (range)	NGNA mol/mol mean (range)
HLX03	72.9	2.5	1.0	21.7	1.1	0.049	ND
	(71.1–75.1)	(2.2–2.9)	(1.0–1.1)	(19.8–23.0)	(1.0–1.2)	(0.044–0.055)	(ND-0.001)
CN-Humira®	71.0	8.0	1.2	18.1	1.1	0.011	ND
	(67.8–72.9)	(6.7–8.4)	(0.9–1.8)	(16.3–22.7)	(0.9–1.4)	(0.007–0.014)	(ND-0.001)

*represent G0F, G0F-GN, G0FB type N-glycans

§represent Man5, Man6, Man7, Man8, Man9 type N-glycans

†represent G1FS1, G1FS1-GN, G2FS1, G2FS2 type N-glycans

‡represent G1, G1’, G1F, G1F’, G1F-GN, G1FB, G2, G2F type N-glycans

**represent G0, G0-GN, G1, G1’, G2 type N-glycans

### Analytical Similarity Comparison of Biological and Immunological Activities of HLX03 to the RP

**Fig. 4:**
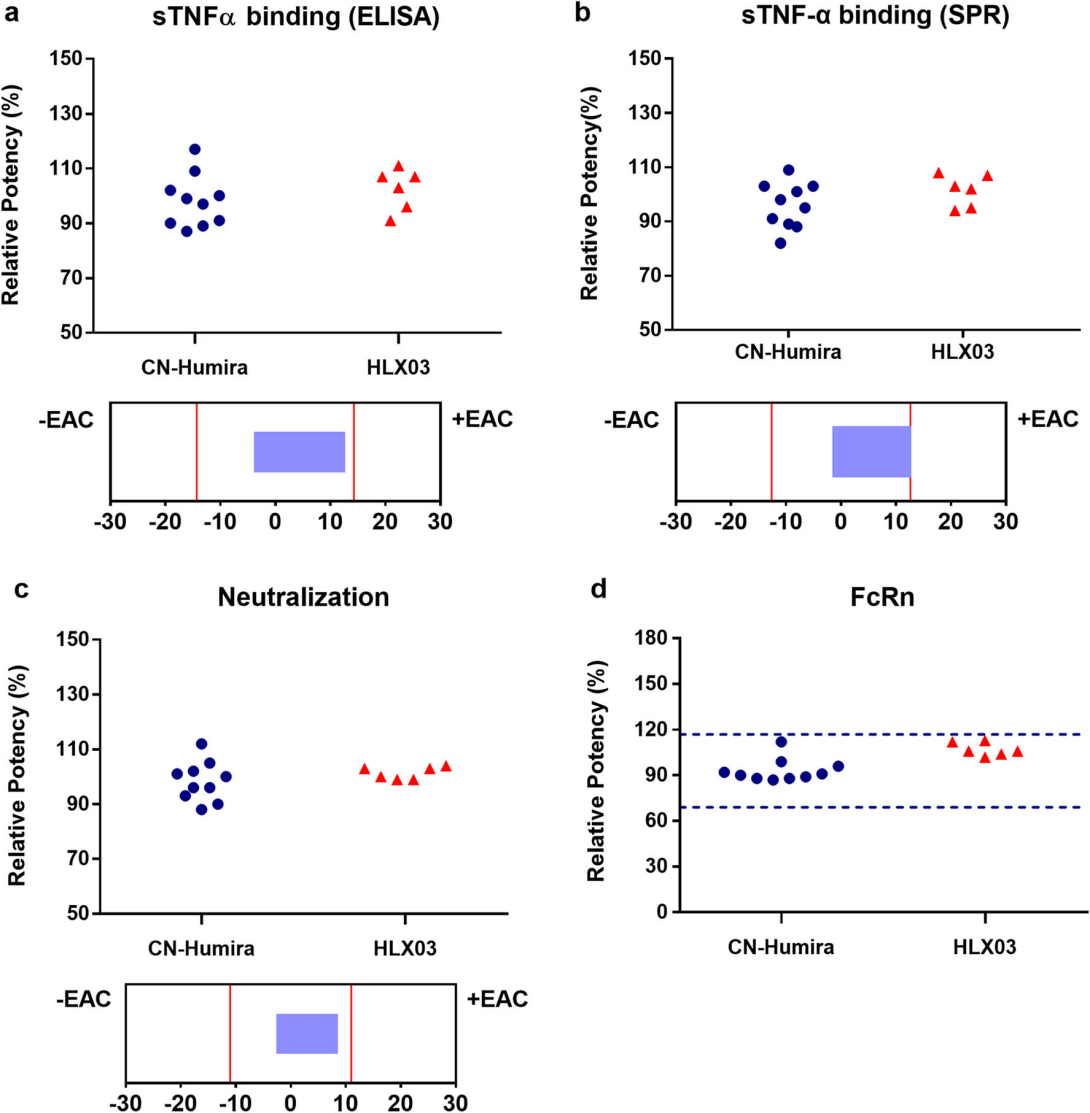
Comparison of tier 1 and tier 2 biological quality attributes between HLX03 and CN-Humira®. Equivalence test results of tier 1 quality attributes with 90% CI plotted under corresponding scatter plot. **a** sTNF-α-binding activity assayed by ELISA, **b** sTNF-α-binding kinetics assayed by SPR, **c** Neutralization activity, **d** FcRn-binding affinity analyzed in tier 2 attribute criteria, quality range (mean ± 3SD)

**Fig. 5:**
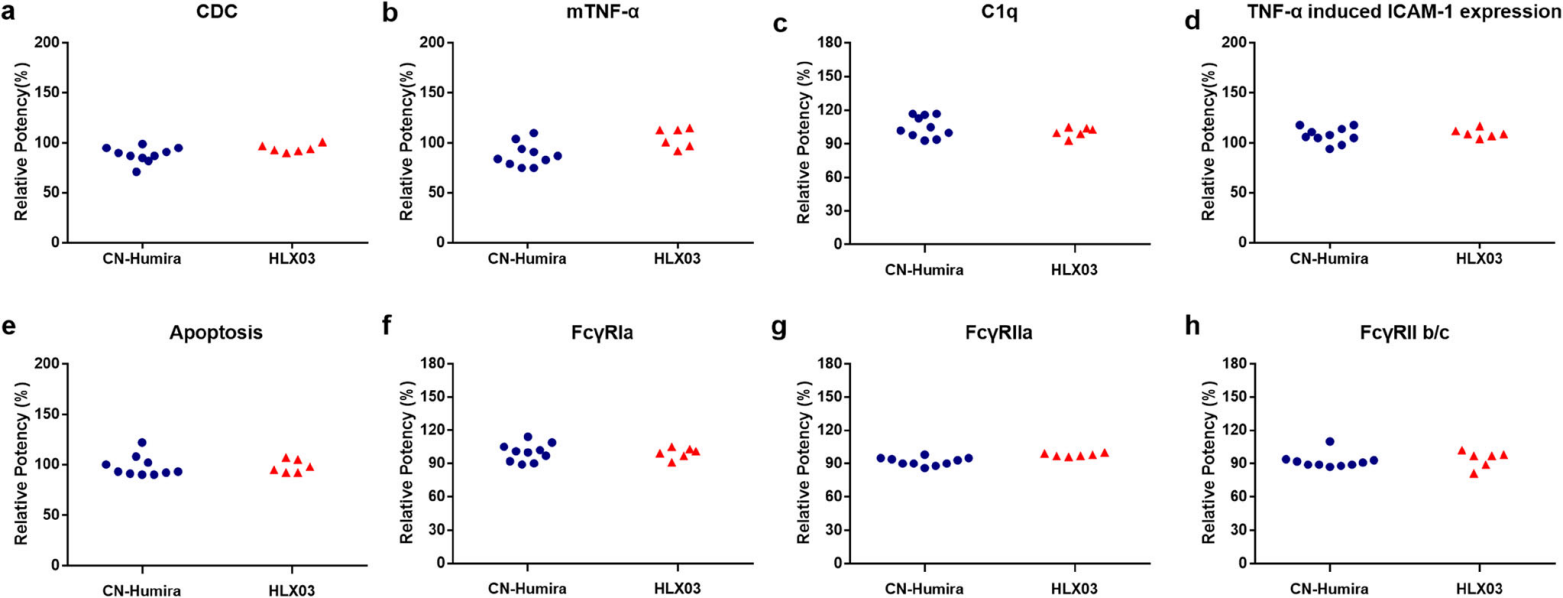
Comparison of tier 3 functional quality attributes between HLX03 and CN-Humira®. The dot plots of **a** CDC activity, **b** tmTNF-α-binding affinity, **c** C1q-binding affinity, **d** TNF-α induced ICAM-1 expression, **e** apoptosis activity, **f** FcγRIa-binding affinity, **g** FcγRIIa-binding affinity, **h** FcγRIIb/c-binding affinity

A battery of sensitive bioassays that reflect adalimumab’s functions were applied in the analytical similarity assessment. Being directly associated with the primary MOA, sTNFα binding, and neutralization potencies were assigned and assessed as tier 1. The sTNF-α binding and the binding kinetics of HLX03 met the statistical equivalence criteria and showed high similarity to the RP (Fig. 4a–c). Neonatal Fc receptor (FcRn)–binding activity, a tier 2 property impacts the clearance of antibody, was similar among all the products (Fig. 4d). Except for FcγRIII binding and ADCC, all the tier 3 functional properties (Table II) including tmTNFα binding, CDC, apoptosis, TNFα, or LTα-mediated ICAM1 expression were visually similar (Fig. 5).

**Fig. 6:**
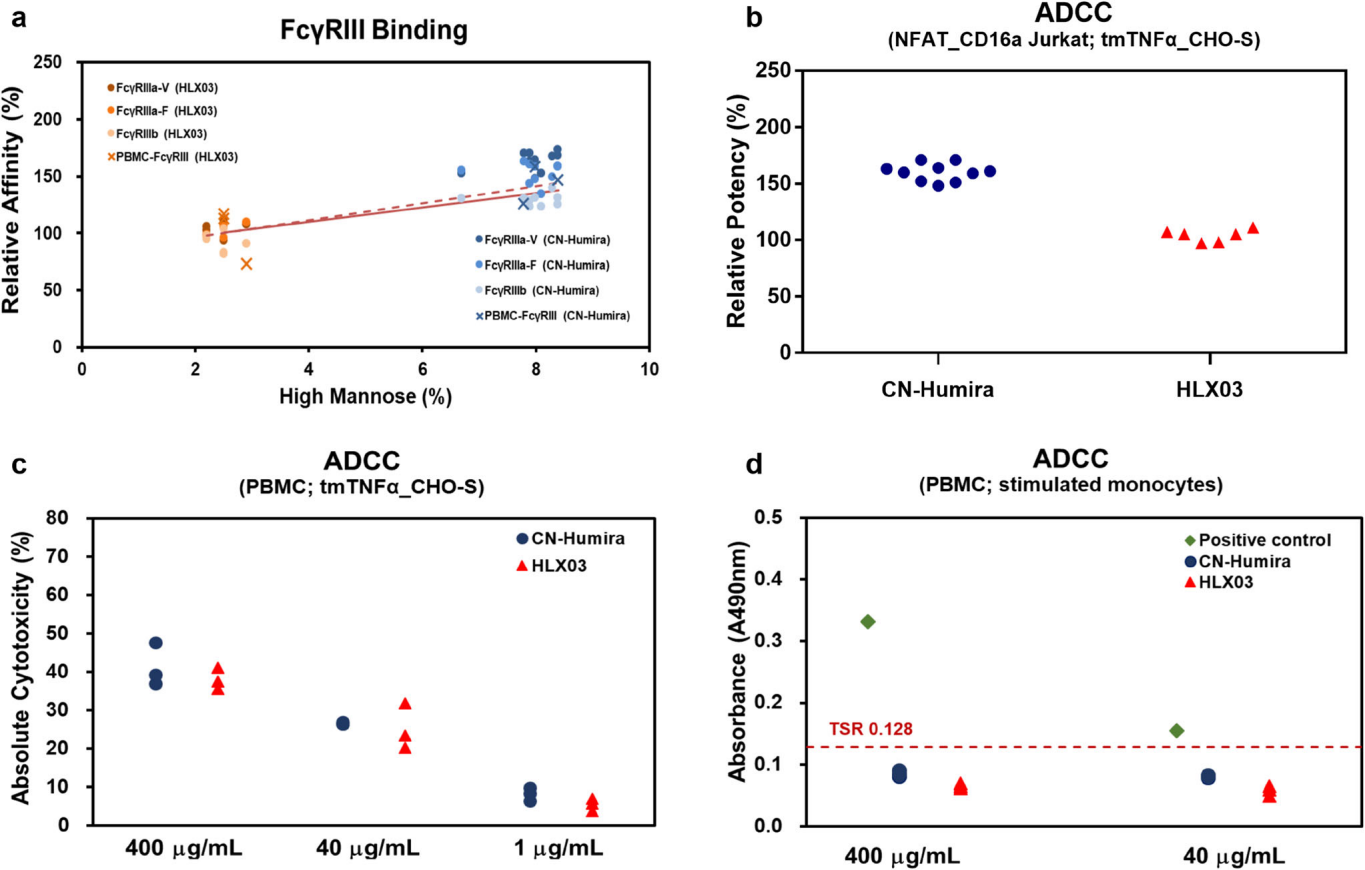
Comparison of FcγRIII-binding affinity and ADCC activity (tier 3) of HLX03 and CN-Humira®. **a** Correlation between %HM- and FcγRIII-binding affinity for HLX03 and CN-Humira®. The dots represent the affinities to recombinant human FcγRIIIs detected by SPR, and the dash line is the corresponding trend line. The × shape represents affinities to the PBMC cell surface FcγRIIIs detected by FACS, and the solid line is the corresponding trend line. **b** ADCC activity assayed by reporter gene method, **c** PBMC-based ADCC activity on tmTNFα overexpressed engineering cell line and **d** PBMC-based ADCC activity on LPS stimulated monocytes. Green diamonds represent positive control, and target spontaneous release (TSR) is served as low control

FcγRIII-binding affinity and ADCC activity, the other two tier 3 functional properties, were visually similar in clinically representative assay based on PBMC cells, although they were slightly lower in HLX03 in the standard protein or genetically engineered cell line–based assay (Fig. 6). SPR is an extremely sensitive method to detect the molecular interaction between immobilized recombinant human FcγRs and mAbs by tracking the change of signal via sensor chips. Despite the slightly lower FcγRIII affinity of HLX03 was observed by SPR testing, there was no significant difference between HLX03 and the RP in the affinity to the FcγRIII on the surface of PBMC (Fig. 6a). In the reporter gene assay, HLX03 exhibited lower ADCC activity than the RP (Fig. 6b), both the FcγRIIIa on effector cells and tmTNF-α on target cells are over expressed, as a result, the ADCC activity is much higher than the normal level in human body. The use of PBMC as effector cells and tmTNFα_CHO-S cells (engineering cell line with over-expressing tmTNF-α) as target cells for ADCC assay can better reflect the similarity between HLX03 and the RP *in vivo*. Figure 6c showed similar ADCC activity of HLX03 and the RP at high, medium, and low antibody concentrations. In addition, neither HLX03 nor the RP exhibited ADCC activities in a clinically representative experimental design using lipopolysaccharides (LPS) stimulated monocytes as target cell and PBMC as effector cell, due to shortage of tmTNF-α in human cells.

In conclusion, all the investigated biological and immunological activities of HLX03 are similar to those of the RP. The PBMC-mediated cytotoxicity of HLX03 with three different concentrations was similar to that of the RP. All these data indicated that the %HM difference between HLX03, and the RP has no effect on FcγRIIIa affinity and ADCC activity *in vivo*.

## Discussion

QbD has become an integral part of therapeutic protein development to obtain high and constant product quality with less risk of batch failures. However, effective implementation of QbD is still a challenge in the biopharmaceutical industries due to complex relationships among quality, efficacy, and process procedures. Here, the similarity assessment of adalimumab biosimilar HLX03 to CN-Humira® demonstrated an example of QbD-based analytical similarity study for biosimilar development.

QbD quality study information was conveyed as a story from QTPP, CQAs, to similarity assessment, and acceptance criteria for each CQA. Although there is no definitive request of QbD information from the regulatory agencies (24), sequential description of QTPP, quality study, CQA, and similarity assessment would be helpful to give the review staff a clear picture of the product. In this study, publicly available information of Humira® and the RP characterization data were first collected to generate the QTPP list of adalimumab biosimilar HLX03. The quality attributes were then thoroughly analyzed using the qualified or verified analytical methods. Since antibodies are a homologous class of molecules, knowledge gained from prior experience or published studies may greatly aid in identification of CQAs (25). The nature of TNFα, patient population, indications, and effector functions were all considered in the risk assessment. The identified CQAs were closely investigated using multiple orthogonal and advanced analytical methods. Based on the risk ranking, quality attributes were assigned to corresponding tiers of 1 to 3 for similarity assessment.

Physicochemical and functional tests demonstrated high similarity of HLX03 to CN-Humira®, except for slightly lower %HM in HLX03. As previously reported (26,27), %HM affects Fc-related bioactivities and half-life of mAbs. Since Fc-related bioactivities have little contribution to the clinical effect on target indications of HLX03 (12), and the %HM difference between HLX03 and the RP was not expected to affect clearance of mAb, HM was identified as a none-CQA for HLX03. Batch to batch %HM variations are existed in the RPs as well. According to the data released by AbbVie (28), the %HM (M5 + M6) ranged from 5 to 8% in the RP batches produced from 2001 to 2013. Meanwhile, the %HM ranged from 6.8 to 9.7% in the EU and US sourced Humira® recorded in an Amgen’s biosimilar paper (29). HM enhances ADCC activity via FcγRIII binding (26). In the most sensitive ADCC experiment system incorporating FcγRIIIa over-expressed effector cells to the tmTNF-α over-expressed target cells, lower ADCC activity was observed for HLX03. In consistent with a previous study (26), the FcγRIII affinity and ADCC activity increase with the increased %HM in the *in vitro* study using recombinant proteins or engineering cell lines. While HLX03 showed similar ADCC to the RP in the experiment using PBMC as effector cells to simulate the *in vivo* condition. The extracellular region of tmTNF-α can be quickly hydrolyzed to soluble TNF-α *in vivo*, leading to a shortage of tmTNF-α in human cells. Further experiments showed that neither HLX03 nor the RP could initiate ADCC activities in the experimental system without using the engineering cell line as the target cell. The ADCC was reported to have no effect on efficiency and safety of the antibody in RA, PA, and Ps treatments (12). Certolizumab pegol, a humanized F(ab’) IgG1 fragment against the TNFα with no ADCC and CDC activities, has consistent efficiency and safety in RA with other TNFα inhibitors (30). The ADCC activities in NK92-CD16a cells of US and EU licensed infliximab (another TNFα inhibitor) were changed along with corresponding afucose plus HM content ranged from 5.6–12.8%, but the ADCC activities were similar for all the products in PBMC (31). All above demonstrated that the %HM difference between HLX03 and the RP would not affect the similarity of ADCC effect in clinical trials. HM can affect the half-life of the mAbs through binding with mannose receptors (27). However, the % HM from 4 to 17% only resulted in 1.0 to 6.0% differences on the area under curve (AUC) in Goetze AM’s study (27). Accordingly, the %HM differences between HLX03 and the RP might have no significant effect on half-life. Furthermore, HLX03 demonstrated pharmacokinetics equivalence and comparable safety with the RP in the phase I clinical trial (32). Preclinical and phase III clinical data also provided similar evidences for similarity of HLX03 to the RP (data unpublished). All above confirmed that the %HM difference did not impact clinical similarity of HLX03 to CN-Humira®. Based on the observations, it can be expected that, other than RA, PA, and Ps, HLX03 will still be demonstrated equivalence to the RP in clinical trial of inflammatory bowel diseases, for which ADCC may has potential functions and would be assigned as tier 2 quality attribute for similarity assessment (33,34).

## Conclusion

Physicochemical and functional tests demonstrated high similarity of HLX03 to CN-Humira®, illustrating an example of QbD-based analytical similarity assessment for a biosimilar mAb development.
